# Agreement between pre-exercise screening questionnaires completed online versus face-to-face

**DOI:** 10.1371/journal.pone.0199836

**Published:** 2018-06-26

**Authors:** Lynda Norton, Jessica Thomas, Nadia Bevan, Kevin Norton

**Affiliations:** 1 Health and Exercise Science, Flinders University, Bedford Park, South Australia; 2 South Australian Health and Medical Research Institute, Adelaide, South Australia; 3 School of Health Sciences, University of South Australia, Adelaide. South Australia; Leibniz Institute for Prevention Research and Epidemiology BIPS, GERMANY

## Abstract

**Objectives:**

To investigate the levels of agreement between self-reported responses to the Adult Pre-exercise Screening System (APSS) questionnaire using online versus face-to-face (F2F) modalities.

**Design:**

Convenience sample of adults completing a pre-exercise screening questionnaire using different modalities.

**Methods:**

Adult volunteers (n = 94) were recruited to complete the APSS using both online and F2F modalities. Participants were provided a URL link to an online APSS questionnaire then followed-up the next day in a F2F interview. Objective health risk factors were also measured. Comparisons between responses were undertaken using kappa and correlation statistics to determine levels of agreement.

**Results:**

The levels of agreement between online versus F2F responses for the seven compulsory Stage 1 questions (known diseases and signs and/or symptoms of disease) were >94% (kappa = 0.644–0.794). Response comparisons for Stage 2 questions on health risk factors were also generally high (>82% agreement) but there were larger differences between reported and measured risk factors in Stage 3.

**Conclusions:**

Levels of agreement between the Stage 1 responses were substantial and support the use of this online option for pre-exercise screening. There were larger differences between self-reported and objectively measured health risk factors in Stages 2 and 3.

## Introduction

Regular participation in moderate, and especially vigorous, intensity physical activity (PA) has been shown to lower the risk of cardiovascular disease (CVD) and CVD mortality in numerous longitudinal and interventional studies [1]. Paradoxically, the risk of cardiovascular or cerebrovascular events (such as myocardial infarction, sudden cardiac death or stroke) is increased during an acute bout of PA, although less so in those who are habitually physically active [2]. The risk of an adverse cardiovascular event accelerates as the relative intensity of PA increases [3]. Typically, known or occult CVD is the most prevalent underlying pathology precipitating PA-associated myocardial infarction and sudden cardiac death in adults aged >35 years [4]. Therefore, efforts to identify adults at increased risk generally focus on screening for medical history of established CVD, respiratory or metabolic diseases, symptomatology and major risk factors of these diseases [5].

A recent study that applied the American Heart Association/American College of Sports Medicine (AHA/ACSM) pre-participation screening to the National Health and Nutrition Examination Survey (NHANES) database found that up to 90% of US adults aged >40 years may receive a recommendation for a medical review prior to commencing a new PA program [6]. It was argued this excessive referral might present an unnecessary barrier to exercise adoption and add substantially to the healthcare budget. These concerns have, in part, driven recent changes to both the AHA/ACSM pre-participation [7] and the Australian pre-exercise screening guidelines [8, 9].

The Australian Adult Pre-Exercise Screening System (APSS) https://www.essa.org.au/for-gps/adult-pre-exercise-screening-system/ was co-developed by an expert panel from Exercise and Sport Science Australia (ESSA), Fitness Australia (FA) and Sports Medicine Australia (SMA) in 2011 [9]. The APSS has been promoted throughout the health and fitness industry to identify individuals who may be at higher risk of experiencing an adverse event when they either first commence a PA program or significantly upgrade their exercise program. High-risk individuals are recommended to seek medical or other health professional guidance before commencing their exercise program. Low- and moderate-risk individuals, even those with health risk factors are encouraged to begin light to moderate PA (such as walking), without referral to a health professional. The APSS was designed by these organisations to facilitate clients completing the questionnaire either online without direct guidance, or in a face-to-face (F2F) scenario. In a sample survey of 1178 exercise professionals conducted approximately 18 months after the APSS release, it was found that 55% were frequently using the APSS [10]. However, the level of agreement of this screening tool when completed using different modalities has not been investigated. It has been previously shown that different modes of data collection can result in variations in responses and rates particularly when dealing with health issues and other sensitive behaviours [11, 12]. The advantages of F2F questionnaires are that they are clearly structured, interaction is personal, questions can be clarified and detailed answers can be provided. On the other hand, there are also some disadvantages such as cost, interviewer bias and social desirability bias. Online surveys are less expensive and easier to administer, they do not require interviewers to be present and participants can complete them in their own time. However, they do require Internet access and participants do not have the facility to clarify questions if they are unsure [12]. Despite the fact that some pre-exercise screening questionnaires are readily available online [13], the authors are not aware of any previous studies that have examined the level of agreement between different modes of administering these questionnaires. The concerns then become: are there systematic differences in responses between the two modes and also how much accuracy are we sacrificing for the sake of expediency?

This study examined the levels of agreement between responses to the APSS questions when completed online versus responses provided to an exercise professional in a F2F setting.

## Methods

The study involved adult participants aged 18–75 years who were recruited through advertising fliers located at a local fitness centre and amateur sports club in 2016. The study was approved by the Human Research Ethics Committee of Flinders University (HREC code 470.14).

Interested volunteers were sent an information package and consent form by electronic or traditional mail. Upon receipt of completed consent, a follow-up appointment was scheduled, by phone, for a pre-exercise screening test and participants were also provided a URL link to an online APSS questionnaire (http://www.fitnessriskmanagement.com.au/screening-tool/). They were asked to complete the self-report sections (Stages 1 and 2) of the APSS the day prior to their scheduled test. Online, participants entered essential demographic information including date of birth, gender and postcode before beginning the APSS. The first stage of the APSS involves seven questions to identify any established cardiovascular, metabolic or respiratory diseases, signs and symptoms of these diseases or other medical issues that represent a substantial risk when beginning or upgrading PA patterns. The professional organisations that developed the APSS recommend this screening stage is compulsory for those joining fitness/gym facilities [9].

The second stage includes self-reported information on major health risk factors for disease or other conditions that may be exacerbated by exercise including information on family history of CVD, smoking status, PA patterns, height, body mass, known hypertension, high cholesterol and/or high blood glucose, hospitalisation in the past 12 months, prescribed medications, pregnancy or recent childbirth, and musculoskeletal symptoms. The online responses of the participants were saved automatically in the online APSS database. The data was exported from the database as an XL spreadsheet and imported into Statview for data linkage to the F2F measures.

Participants undertook their pre-exercise screening at the University the day following the completion of the online survey and having fasted for at least eight hours. The APSS questionnaire was again administered, this time in a F2F setting, by a researcher blinded to their previous online responses. In addition to the Stage 1 and 2 questions, Stage 3 measurements included resting blood pressure taken after at least five minutes of being seated using a Dinamap Pro 100 sphygmomanometer, anthropometric measures of height, mass and waist girth following the techniques of Norton and colleagues [14] and total cholesterol and blood glucose recorded using a Reflotron analyser [15].

The additional information obtained as part of Stages 2 and 3 is optional in the APSS system but may be used to refine the individual’s health risk profile prior to exercise prescription or to identify extremes in health risk factors. Data linkage between the online database and F2F responses was possible using demographic details. Database access was limited to the researchers and password protected.

Comparisons were made between responses in the following way: Intra-rater responses to dichotomous questions in stage 1 were evaluated using the overall level of agreement and the kappa statistic. Continuous variables from stages 2 and 3 were analysed using intra-class correlations and paired t-tests. Skewed PA data were analysed using Spearman correlation analysis and Mann-Whitney tests. All statistical analyses were carried out using Excel spreadsheets and Statview statistical software (Abacus Concepts Inc, Berkeley, CA, USA). Kappa values of 0.81 and above represented almost perfect agreement, and values 0.61–0.80, 0.41–0.60, 0.21–0.40 and 0–20 represented substantial, moderate, fair, and slight agreement, respectively [16]. The sample size for this study to allow the detection of a kappa statistic of 0.40 with 80% power at an alpha level of 0.05 was at least 50 participants [17].

## Results

Ninety-four participants took part in the study and their details are shown in Table 1. The table includes a range of both self-report and measured values for anthropometry, PA behaviours and blood parameters. The numbers of participants classified as high, moderate or low risk calculated for the two modes are also shown. Supplementary data is provided in the Supporting Information file S1 Data Table.

**Table 1:** Descriptive data for major health risk factors and physical activity behaviours for the 94 participants based on the APSS. Values are shown as mean (± standard deviation); and/or median (± inter-quartile range).

Variable	Self-report (online)	Measured (F2F)
	Mean (±SD)	
Age (yr)		42.6 (±15.7)
Gender (% males)		53.2
Systolic blood pressure (mmHg)		130 (±16)
Diastolic blood pressure (mmHg)		80 (±9)
Fasting blood glucose (mM)		5.29 (±0.92)
Total cholesterol (mM)		4.65 (±1.06)
Height (cm)	172.7 (±8.7)	172.0 (±14.0)
Mass (kg)	75.7 (±18.5)	76.2 (±18.8)
Calculated Body Mass Index (kg/m^2^)	25.3 (±5.9)	25.4 (±6.2)
Waist girth (cm)		82.2 (±12.7)
Hip girth (cm)		98.9 (±9.3)
Waist-hip ratio		0.83 (±0.09)
**Physical Activity**	**Self-report (online)**	**Self-report (F2F)**
	Mean (±SD); median (IQR)	
Walk (number of times/wk)	7.6 (±7.1); 5 (±7)	7.0 (±5.2); 5 (±5.8)
Walk (min/wk)	137 (±137); 95 (±120)	149 (±129); 120 (±140)
Moderate (number of times/wk)	1.8 (±2.6); 1.0 (±3)	2.0 (±5.4); 1 (±3)
Moderate (min/wk)	77 (±156); 30 (±92)	71 (±1067); 25 (±120)
Vigorous (number of times/wk)	4.4 (±3.9); 4.0 (±4)	4.0 (±3.4); 3 (±4)
Vigorous (min/wk)	201 (±189); 145 (±255)	191 (±187); 120 (±195)
Total weighted physical activity (min/wk)	621 (±471); 530 (±589)	602 (±423); 530 (±550)
Risk category (high, mod, low)	28, 4, 62	21, 16, 57

### Physical activity behaviour

Table 1 shows when the PA patterns were compared, vigorous intensity exercise showed a strong correlation for both the number of sessions per week (Rho = 0.92) and minutes of PA per week (r = 0.89). Greater variation between self-reporting modalities was identified when comparing moderate intensity PA resulting in a lower ICC (Rho = 0.67, and r = 0.34, respectively). Walking patterns showed an intermediate correlation (Rho = 0.76, and r = 0.64, respectively). Paired t-tests showed none of these intensity comparisons for PA time per week were different (p = 0.27, 0.51 and 0.18 for vigorous, moderate and walking, respectively). Similarly, Mann-Whitney tests showed the number of sessions per week at different intensities were not different (p = 0.94, 0.59 and 0.47, respectively). When the total number of PA sessions (vigorous + moderate + walking) was compared, participants reported slightly more PA sessions online versus F2F (13.5 versus 12.8 per week, p = 0.04).

Table 2 shows the kappa statistic for Stage 1 questions when self-reporting CVD events, chest pain, fainting or dizziness, muscle joint pain and other medical conditions between online and F2F modes were all within the substantial agreement category. Levels of agreement for these questions ranged between 94% and 99% of responses. Kappa values could not be calculated for the questions concerning ‘medical attention for an asthma attack’ or ‘poorly controlled diabetes’ because some cells had a zero frequency (questions 4 and 5 of the APSS). However, there were very high levels of agreement between the modalities in responses to these questions. The results in Table 2 showed the frequency of ‘yes’ responses was higher for all Stage 1 questions answered online when compared with the F2F setting.

**Table 2:** Results of the comparisons between the online and F2F responses and other measured variables. Stages 1 and 2 are online versus F2F responses. * Total weighted physical activity = walking + moderate + (2x vigorous minutes). ** No pregnancies were reported in either mode. *** Stage 3 relates to measured health risk factors versus online responses (except waist). Y = Yes; N = No; CVD = cardiovascular disease; SBP = systolic blood pressure; DBP = diastolic blood pressure.

**Stage 1 known disease / signs & symptoms**	**n**	kappa	95% CI	ICC (RMS)	Agreement (%)	Self-report [online] = YES	Self-report [F2F] = YES	Self-report [online] = NO	Self-report [F2F] = NO
Qu (1) Heart condition or stroke [Y or N]	94	0.644	0.321–0.964		96	7	5	87	89
Qu (2) Chest pain [Y or N]	94	0.752	0.483–1.020		97	7	6	87	88
Qu (3) Fainting / dizziness [Y or N]	94	0.653	0.290–1.015		97	6	3	88	91
Qu (4) Asthma [Y or N]	94				97	3	0	91	94
Qu (5) Diabetes [Y or N]	94				99	1	0	93	94
Qu (6) Muscle, bone, joint problem [Y or N]	94	0.733	0.531–0.935		94	14	12	80	82
Qu (7) Other medical [Y or N]	94	0.794	0.402–1.186		99	3	2	91	92
**Stage 2 Self-report risk factors**	** **								
Age (M ≥ 45; F ≥ 55yr)	94					36		58	
Family history [CVD] [Y or N]	89	0.753	0.486–1.019		97	12	13	77	76
Smoker [current or quit last 6 months]	93	0.884	0.658–1.109		99	3	2	90	91
*Calculated physical activity [total min/wk]	94			0.819 (272)					
*Calculated physical activity [<150 min/wk]	94	0.733	0.512–0.955		95	12	9	82	85
Height [cm]	84			0.982 (1.6)					
Mass [kg]	84			0.994 (1.8)					
Calculated Body Mass Index [kg/m^2^]	91			0.974 (0.98)					
Calculated Body Mass Index [≥30kg/m^2^]	83	1.000			100	6	6	77	77
High blood pressure [Y or N]	92	0.776	0.565–0.988		96	9	11	83	81
High cholesterol [Y or N]	92	0.888	0.879–1.039		99	14	15	78	77
High blood glucose [Y or N]	92	0.554	0.105–1.003		97	3	4	89	88
**Stage 2 other questions									
Hospitalisation [Y or N]	91	0.263	0.000–0.597		90	9	4	82	87
Musculoskeletal pain/soreness [Y or N]	93	0.469	0.255–0.683		82	23	19	69	75
***Stage 3 Measured risk factors	**n**	kappa		ICC (RMS)	Agreement (%)	Self-report [online] = YES	Measured = YES	Self-report [online] = NO	Measured = NO
Height [cm]	91			0.949 (2.6)					
Weight [kg]	91			0.981 (2.4)					
Calculated Body Mass Index (≥30)	91	0.776	0.565–0.984	0.962 (1.3)		8	12	83	79
Waist girth [M >94; F > 80 cm]	84						20		64
High blood pressure [SBP≥140 or DBP≥90mmHg]	91	0.228	0.019–0.438		75	10	25	81	66
High total cholesterol [≥5.2mM]	68	0.123	0.000–0.344		66	5	23	63	45
High blood glucose [>5.5mM]	46	0.062	0.000–0.190		62	1	28	45	18
**Risk stratification**	** **								
Risk category [high]		0.644	0.469–0.818		86	28	21	66	73

The kappa statistic and ICC values for response comparisons in Stage 2 of the APSS indicate substantial to near-perfect agreement was found in the reporting of all health risk factors with the exception of the question ‘have you been told that you have high blood sugar?’ which had a kappa in the moderate agreement category. The correlation coefficients for self-reported height, weight and calculated Body Mass Index (BMI) between online and F2F modes were very high, ranging between 0.97 and 0.99. The questions related to musculoskeletal and hospitalisation achieved fair–moderate kappa values. Table 2 shows a strong correlation in reported total PA time and a substantial level of agreement in the calculated PA for PA-related health risk (<150 min/week).

In Stage 3 of the APSS correlations were very high between self-report and measured anthropometry variables. There were substantial differences when comparing online self-reporting to objectively measured health risk factors. The online responses indicated lower agreement rates (%) than objectively measured risk levels for high blood glucose (62%), cholesterol (66%) and blood pressure (75%). The incidence of measured health risk factors is greater than when self-reported. The largest discrepancies were found in the measured risk factors of blood pressure, total cholesterol and fasting glucose and resulted in slight-fair kappa values. The differences between the two self-report modes (Stage 2 questions) were not as extreme and were also underreported online, relative to F2F. The kappa values ranged between fair-substantial.

## Discussion

The APSS seeks to reduce the risk of injury and adverse events while encouraging greater levels of PA and associated health benefits for exercise participants. The current study used two different modalities of administration of the APSS to determine the levels of agreement between responses. In addition, measurements of several health risk factors were included as part of the APSS Stage 3 and comparisons made between reported and measured risk rates. Based on kappa statistics and correlation analysis the levels of agreement between the seven compulsory Stage 1 questions completed online versus F2F were very high. Response comparisons for Stage 2 questions were also generally high but there were larger differences between reported and measured health risk factors in Stage 3.

Screening prior to beginning or upgrading an exercise program is important because it can help stratify participants for relative health risk and assist in tailoring exercise to individual capabilities. It also forms part of the provider’s legal duty of care to those starting programs [18] and is included in health and fitness curricula as a professional standard [19]. Despite this, recent national surveys have indicated 24% of exercise/gym facilities in the USA [20] and 35% in Australia [10] were not using any pre-participation screening of new members.

These statistics and others relating to the reported high exclusion rates when undertaking pre-exercise screening, together with questions concerning modes of administration [8, 21, 22] have stimulated efforts to develop more accessible and reliable tools for screening [7, 9, 13]. This has included using contemporary methods such as online self-reporting as well as a trend towards more liberal approaches when dealing with a range of health risk factors and chronic conditions. For example, encouraging more people to undertake low-moderate intensity PA, such as walking, without the need for medical review. The overall direction is to place greater emphasis on the exercise professional to prescribe appropriate PA and to monitor an individual’s tolerance and progression. The APSS tool was developed to be practical, sensitive and easy to use in a range of settings in Australia including guidance when self-administered [9].

Stage 1 of the APSS is a critical part of the screening process because it reveals details of previous diseases, and/or signs and symptoms of major diseases. These can present serious, albeit rare, challenges during unaccustomed exercise and this is why it has been deemed the compulsory stage. However, there is a balance required between completing this stage of the APSS online for convenience and encouraging more PA versus recommending F2F interviewing for all clients. The current APSS model represents the decision struck after taking into consideration elements such as the low relative risk of adverse events during exercise, and the additional time and expense of more intensive interview-based approaches.

Stage 1 showed a comparatively low rate of ‘yes’ responses to the compulsory questions concerning known and/or signs and symptoms of disease, ranging between 1–15% (Table 2), despite large age and previous PA ranges. The substantial agreement levels between the two survey modes are encouraging. Importantly, clients answering online were more likely to answer ‘yes’ than when compared to their responses in the F2F interview scenario, for all seven questions. This pattern is typical for self-administered online health surveys, where respondents are more likely to report poorer health when compared to F2F interviewing [11]. Further analyses, however, indicate the need to be cautious given there were four individuals where the initial online response was ‘no’ but the subsequent F2F response was ‘yes’ resulting in a high risk classification (two for CVD and two for musculoskeletal problems). This discrepancy, together with approximately 30% of online participants being classified as high risk, reinforces the importance for exercise professionals to clarify responses [21, 22]. This was also highlighted in a review of the updated PAR-Q screening questionnaire, called the PAR-Q+ system [13, 23]. The PAR-Q+ reduced the number of people who were referred for medical evaluation prior to beginning an exercise program (relative to the original PAR-Q). While slightly more people were identified using the seven general health questions (22 vs 15%), the introduction of a series of mandatory follow-up questions, including a more detailed online questionnaire (ePARmed-X+) and a review provided by a qualified exercise professional, reduced the proportion of medical referrals to approximately 1% of the original cohort [13]. However, this comes at a cost since their definition of a ‘qualified exercise professional’ is a ‘qualified university educated fitness professional’ with ‘advanced education, training and certification’ [13].

Completing the APSS compulsory screening questions online presents a quick, convenient and less resource-intensive option but it is not infallible. Previous research has shown it is impossible for population-level, self-reporting of health-related information to be 100% reliable, no matter what the delivery mode [13, 24]. Therefore, it should not be expected that the APSS would show 100% agreement even in the compulsory stage of the screening process although this study showed it to be close.

Stage 2 is an optional level of screening designed to identify participants with health risk factors or conditions that assist with appropriate exercise prescription, although it can also be used to identify extremes or unusual combinations in risk factors that may then require further professional guidance. Stage 2 is self-reported and was developed to assist qualified professionals prescribe tailored exercise programs.

The results showed most questions had at least a substantial level of agreement between the online and F2F responses (Table 2). The question concerning ‘have you ever been told you have high blood glucose’, showed a moderate level of agreement. Lower levels of agreement were found for the questions concerning ‘recent hospitalisation’ and ‘musculoskeletal pain or soreness made worse by PA’. In both questions there were higher levels of positive responses online compared to F2F. The broad nature of these two questions is likely to capture more positive responses online than when delivered in a F2F setting as has been reported for other sensitive health information [11, 12]. More importantly, the utility of these two questions is based on clarification of positive responses by a qualified exercise professional [9]. Without follow-up probing of positive responses the tailoring of PA may be sub-optimal. This approach is formalised in the case of the PAR-Q+ for those indicating bone and/or joint problems [23].

The optional, Stage 3 screening is designed to obtain objective measures of CVD and metabolic risk factors. These can be used to refine the individual’s health risk profile prior to prescribing exercise. All measured risk factor rates were higher than self-reported levels. A relatively small number of participants under-reported BMI risk. However, there were large discrepancies in high blood pressure, total cholesterol and fasting glucose rates. While the precise reasons are unknown this inconsistency may be due to either social desirability bias or, more likely, participants being unaware they had a risk factor. Recent Australian Health surveys have shown that approximately 90% of adults with high cholesterol and 70% with high blood pressure were unaware of these risks [25, 26]. This limitation has been identified with previous screening protocols [21] and the APSS system now tempers this by encouraging light-moderate PA even for those with health risk factors (whether known or not).

The generalisability of this study may be limited firstly by the small sample size, and by a healthy participant bias given that volunteers in the current study were more physically active than comparable general population levels [26]. However, the measured risk factor rates were similar to the reported national rates for adult Australians [25, 26].

## Conclusion

Overall, this study has shown the levels of agreement for self-reporting health information between the online and F2F modes in all compulsory questions of Stage 1 were substantial. However, levels of agreement between the two modes for self-reported health risk factors in Stage 2 were more variable and ranged between fair to substantial. The very large differences between some self-reported and measured risk factor rates in Stage 3 highlight the inappropriateness of relying on self-report to determine risk factor profiles. Operationally, Stage 1 results support the use of the online questionnaire to determine whether further guidance from a health professional is recommended prior to beginning exercise. Differences in reported risk factor rates are managed within the APSS decision-making tree such that light-moderate exercise can still be undertaken with appropriate supervision and progression.

## Supporting information

S1 Data Table (XLSX)
